# Lipoxin Inhibits Fungal Uptake by Macrophages and Reduces the Severity of Acute Pulmonary Infection Caused by *Paracoccidioides brasiliensis*

**DOI:** 10.1155/2015/852574

**Published:** 2015-10-08

**Authors:** Laura R. R. Ribeiro, Flávio V. Loures, Eliseu F. de Araújo, Cláudia Feriotti, Tânia A. Costa, Carlos Henrique Serezani, Sonia Jancar, Vera L. G. Calich

**Affiliations:** ^1^Departamento de Imunologia, Instituto de Ciências Biomédicas, Universidade de São Paulo, Avenida Professor Lineu Prestes 1730, 05508-900 São Paulo, SP, Brazil; ^2^Department of Microbiology and Immunology, Indiana University School of Medicine, Indianapolis, IN 46202, USA

## Abstract

Cysteinyl leukotrienes (CysLTs) and lipoxins (LXs) are lipid mediators that control inflammation, with the former inducing and the latter inhibiting this process. Because the role played by these mediators in paracoccidioidomycosis was not investigated, we aimed to characterize the role of CysLT in the pulmonary infection developed by resistant (A/J) and susceptible (B10.A) mice. 48 h after infection, elevated levels of pulmonary LTC_4_ and LXA_4_ were produced by both mouse strains, but higher levels were found in the lungs of susceptible mice. Blocking the CysLTs receptor by MTL reduced fungal loads in B10.A, but not in A/J mice. In susceptible mice, MLT treatment led to reduced influx of PMN leukocytes, increased recruitment of monocytes, predominant synthesis of anti-inflammatory cytokines, and augmented expression of 5- and 15-lipoxygenase mRNA, suggesting a prevalent LXA_4_ activity. In agreement, MTL-treated macrophages showed reduced fungal burdens associated with decreased ingestion of fungal cells. Furthermore, the addition of exogenous LX reduced, and the specific blockade of the LX receptor increased the fungal loads of B10.A macrophages. This study showed for the first time that inhibition of CysLTs signaling results in less severe pulmonary paracoccidioidomycosis that occurs in parallel with elevated LX activity and reduced infection of macrophages.

## 1. Introduction

Paracoccidioidomycosis (PCM) is a chronic systemic infectious disease of Latin America caused by the dimorphic fungus* Paracoccidioides brasiliensis*. The infection is usually acquired by the respiratory route probably by inhalation of airborne propagules [1]. The disease has a wide gamut of clinical and immunopathological manifestations. High antibody titers and suppressed T cell mediated immunity characterize the severe forms of the disease whereas the opposite pattern of immunity is associated with mild PCM [2].

Our laboratory developed an isogenic murine model of paracoccidioidomycosis where A/J and B10.A mice mimic the benign and severe forms of the disease, respectively. A/J mice develop a regressive infection characterized by intense cellular immunity and organized lesions. In contrast, B10.A mice show a progressive disease associated with suppressed T cell immunity and intense tissue pathology caused by fungi-rich disorganized lesions [3, 4]. IFN-*γ* is the main protective cytokine to pulmonary PCM, and IFN-*γ* depletion causes more severe disease in both, susceptible and resistant mice [5]. The innate immunity of these polar mouse strains appears to be paradoxical to the patterns of adaptive immunity they develop. Early in the infection, susceptible mice mount a proinflammatory response mediated by intense IL-12 and nitric oxide production that induce an M1 phenotype in macrophages and DCs, which are able to control the initial fungal growth. In contrast, the innate response of resistant mice is predominantly TGF-*β* mediated, resulting in an M2 profile of macrophages and DCs that are permissive to fungal growth. However, the tolerogenic response of A/J mice is concomitant with the development of immunogenic DCs that secrete high levels of TNF-*α* and induce the activation of protective T cell immunity. In contrast, the excessive proinflammatory innate immunity of B10.A mice induces an early energy of T cells that leads to progressive disease [6–9]. The inflammatory reaction is a complex and multimediated phenomenon that comprises the synergistic action of several mediators such as the eicosanoids derived from arachidonic acid (AA) metabolism. The enzymatic oxidation of AA by cyclooxygenase produces prostaglandins, thromboxanes, and prostacyclins whereas the 5-lipoxygenase (5-LO) is an enzyme that in the presence of FLAP (Five Lipoxygenase Activating Protein) catalyzes the oxidation of AA for the synthesis of leukotrienes (LTs) and lipoxins (LXs). The unstable intermediate LTA4 generated by 5-LO can be converted to LTB4 by enzymatic hydrolysis or, by incorporating the glutathione residue, can be converted to LTC_4_, LTD_4_, and LTE_4_, collectively called Cysteinyl LTs (CysLTs) [10–13].

To date, four LT receptors have been described, B leukotriene receptors 1 and 2 (BLT1 and BLT2) and cysteinyl leukotriene receptors 1 and 2 (CysLT_1_ and CysLT_2_) [14, 15]. The lipoxins are mainly involved in the resolution of inflammatory reactions and are synthesized by two main transcellular routes. One involves platelets/leukocytes interaction and the conversion of 5-LO-derived LTA_4_ into LXA_4_ and LXB_4_ by the enzymatic action of 12-LO. The second pathway is initiated by 15-LO that produces 15*S*-HETE from AA that is rapidly converted to LX by 5-LO [13]. The anti-inflammatory effects of lipoxins are mediated by an LX receptor termed ALX and include inhibition of PMN neutrophils chemotaxis, modulation of cytokines synthesis, and inhibition of cell proliferation [13, 16].

The importance of the LTs as inflammatory mediators and their function in the host defense against infectious agents are well established [11, 12]. In several studies, LTs have been shown to participate in the microbicidal ability of phagocytes, in vivo clearance of pathogens, regulation of cytokine production, and control of the influx of inflammatory cells to the site of infection [17–23]. Although less investigated, the anti-inflammatory lipoxins were also described to play a regulatory role in infections caused by protozoan and bacterial infections [24, 25].

In pulmonary histoplasmosis, LTs were shown to exert a protective role mediated by the control of fungal burden, cytokines production, and the influx of effector and memory T cells into the lungs [26, 27]. The influence of lipid mediators from the AA metabolism was scarcely studied in paracoccidioidomycosis. However, some studies have shown that PGE_2_ impairs the fungicidal ability of macrophages and induces an early immunosuppression in* P. brasiliensis* infected mice [28–30]. Interestingly, an endogenous synthesis of PGE and LTB_4_ by* P. brasiliensis* yeasts was also described, suggesting that fungal cells could use these eicosanoids to modulate host immune responses [31–33]. The role played by LTs in murine paracoccidioidomycosis is still controversial and opposite results showing deleterious or beneficial effects have been recently reported [34–36].

To further elucidate the role of LTs in murine paracoccidioidomycosis, we aimed to investigate the role of CysLTs in the in vitro and in vivo infections caused by* P. brasiliensis*. The influence of montelukast (MTL), an antagonist of the CysLT_1_ receptor, was studied in the acute and chronic phases of pulmonary PCM developed by resistant and susceptible mice. We verified that susceptible mice secreted higher levels of LTs and LXs than resistant mice, and only the former strain developed a less severe early infection when CysLTR_1_ was blocked. The reduced fungal burden induced by MTL treatment was associated with altered migration of inflammatory cells to the lungs, decreased production of proinflammatory cytokines allied with increased levels of IL-10, and typical responses of predominant LX signaling. In vitro studies replicated our in vivo findings: decreased phagocytic activity and reduced fungal loads were recovered when CysLTR_1_ signaling was inhibited. Moreover, addition of exogenous LX reduced the fungal loads, whereas the blockade of LX receptor increased the fungal loads of macrophages. In conclusion, CysLTs and LXs appear to influence innate immunity of* P. brasiliensis* infected hosts. Inhibited CysLTs signaling appears to result in predominant LX activity that reduces* P. brasiliensis* infection. This is an unusual finding, but it adds to others consistently obtained in our model, demonstrating that the uncontrolled activation of the immune system can lead to a more severe PCM [6–9, 37–40].

## 2. Materials and Methods

### 2.1. Ethics Statement

Animal experiments were performed in strict accordance with the Brazilian Federal Law 11, 794, establishing procedures for the scientific use of animals, and the State Law establishing the Animal Protection Code of the State of São Paulo. All efforts were made to minimize suffering, and all animal procedures were approved by the Ethics Committee on Animal Experiments of the Institute of Biomedical Sciences of University of São Paulo (Proc.76/04/CEEA).

### 2.2. Mouse Strains

Unless otherwise stated, groups of 6 to 8 male mice (9 to 11 weeks old) from the susceptible (B10.A) and resistant (A/J) strains to* P. brasiliensis* infection were used for each period of infection. Male 5-LO knockout (KO, 129-Alo5^trnIFun^) and strain matched wild type (WT) SV/129 mice were obtained from the Jackson Laboratory. All the animals were bred at the University of São Paulo animal facilities and provided with acidified water and sterilized food and bedding. All efforts were made to minimize suffering, and all animal procedures were approved by the Ethics Committee on Animal Experiments of the Institute of Biomedical Sciences of University of São Paulo (Proc.76/04/CEEA).

### 2.3. *P. brasiliensis* Infection

*P. brasiliensis* 18 (Pb18) isolate, which is highly virulent, was used throughout this study [41]. Pb18 yeast cells were maintained by weekly subcultivation in semisolid Fava Netto's culture medium [42] at 35°C and used at the 7th day in culture. Mice were anesthetized and submitted to i.t.* P. brasiliensis* infection as previously described [43]. Briefly, after i.p. anesthesia, the animals were infected with 1 × 10^6^, 1 × 10^5^, or 1 × 10^4^ *P. brasiliensis* Pb18 yeast cells, contained in 50 *μ*L of PBS, by surgical i.t. inoculation that allowed dispensing the fungal cells directly into the lungs.

### 2.4. Administration of CysLTR_1_ Inhibitor

Montelukast (MTL, Singular, trade name), a CysLTs antagonist that blocks the action of leukotrienes on the cysteinyl leukotriene receptor-1 (CysLTR_1_), was purchased from Merck Sharp & Dome. For in vivo experiments, MTL was administered via oral route (10 mg/kg/day) in the drink water, and the required drug dilution was prepared immediately before use [44]. MTL treatment started at day −1 of infection and remained for the next two days, when mice were sacrificed. Control mice received equivalent quantities of vehicle in the drinking water. Mice were maintained in individual cages and drug intake monitored by daily differences in water volume. Two treatment schedules were employed for experiments of 8 weeks of infection. One group received 10 mg/kg/day during 11 days, and the other group received the same dose during the whole time of the experiment.

### 2.5. Assay for Organ Colony Forming Units (CFU)

The number of viable microorganisms in the lungs, liver, and spleen from drug-treated and untreated mice was determined by counting the number of CFU. Six to eight animals from each group were sacrificed 48 h and 8 weeks after infection and the enumeration of viable organism in the three organs was done as previously described [45]. Briefly, aliquots (100 *μ*L) of the cellular suspensions of each organ were plated on brain heart infusion (BHI) agar (Difco) supplemented with 4% (vol./vol.) horse serum (Instituto Butantan, São Paulo, Brazil) and 5% Pb192 culture filtrate, the latter constituting a source of growth-promoting factor. The plates were incubated at 35°C and colonies were counted daily until no increase in counts was observed. The numbers (log_10_) of viable* P. brasiliensis* colonies per gram of tissue are expressed as means ± standard errors.

### 2.6. Measurement of Cytokines and NO

The mice were infected i.t. with* P. brasiliensis* and 48 h or 8 weeks later their lungs were aseptically removed and disrupted in 4.0 mL of RPMI 1640 medium (Gibco BRL). Supernatants were separated from cell debris by centrifugation at 2,000 ×g for 15 min, passed through 0.22-*μ*m pore-size filters (Millipore), and stored at −70°C. Cytokines (TNF-*α*, GM-CSF, IL-12, IL-10, IFN-*γ*, IL-2, IL-4, IL-5, IL-17, and IL-23) and chemokine (MCP-1) levels were measured by capture enzyme-linked immunosorbent assay (ELISA) with antibody pairs purchased from BD Pharmingen. The ELISA procedure was performed according to the manufacturer's protocol and absorbance was measured with VersaMax Microplate Reader (Molecular Devices). NO production was quantified by the accumulation of nitrite in the supernatants from in vitro and in vivo protocols by a standard Griess reaction [46]. All determinations were performed in triplicate and expressed as *μ*M NO.

### 2.7. Assessment of Lung Inflammatory Exudates by Flow Cytometry

After 48 h of infection, lungs from each mouse were digested enzymatically for 30 minutes with collagenase (1 mg/mL) in culture medium (Sigma). Lung cells suspensions were centrifuged in presence of 20% Percoll (Sigma) to separate leukocytes from cell debris. Total lung leukocyte numbers were assessed in the presence of trypan blue using a hemocytometer; viability was >85%. The lung leukocytes were resuspended at 10^6^ cells/mL in staining buffer (PBS + 0.1% NaN3 + and 1% fetal calf serum). Fc receptors were blocked by unlabeled anti-CD16/32 antibodies (BD Biosciences) and cells were stained for 20 minutes at 4°C by phycoerythrin-labeled (PE) anti-Dectin-1 and TLR4; fluorescein isothiocyanate-labeled (FITC) anti-MR and TLR2; Peridinin Chlorophyll Protein Complex (PerCP) Cy5.5 anti-CD11b; Pacific Blue (PB) anti-F4/80 monoclonal antibodies (BD Biosciences, eBiosciences). Cells were fixed with 2% paraformaldehyde (Sigma) and stored in the dark at 4°C until analyzed in a flow cytometer FACSCanto (BD Bioscience). The acquisition and analysis gates were restricted to the macrophages using the FlowJo software (Tree Star, Inc.).

### 2.8. Quantitative Analysis of mRNA Expression

After 48 h of infection, lungs from each mouse were removed and RNA was extracted using Trizol reagents. The RNA concentrations were determined by spectrophotometer readings at an absorbance of 260 nm. First-strand cDNAs were synthesized from 2 *μ*g RNA using the High Capacity RNA-to-cDNA kit (Applied Biosystems) according to the manufacturer's instructions. Real-time polymerase chain reaction (RT-PCR) specific for ALOX5 (Mm PT58.30176779), ALOX15 (Mm00507789_m1), NOS2 (Mm00440502_m1), and ARG1 (Mm00475988_m1) (Applied Biosystems, Life Technologies) was performed using the TaqMan real-time PCR assay (Applied Biosystems, Life Technologies) or Sybr Green (Applied Biosystems, Life Technologies) according to the manufacturer's instructions. Analysis was performed with the Stratagene Mx3005P detection system (GE electronics). All values were normalized to GAPDH or HPRT and the relative gene expression calculated.

### 2.9. Leukocyte Subsets in Bronchoalveolar Lavage Fluid (BALF)

Two days after infection, lungs of mice were lavaged after the cannulation of trachea with polyethylene tubing, which was attached on a tuberculin syringe. The same procedure was applied to sham-infected (submitted to surgical stress and injected with 50 *μ*L of PBS) and normal mice of both mouse strains. The lungs were lavaged by repeated injections of 0.5 mL of sterile PBS (final volume, 2.0 mL). The recovered fluid was cytospun at 1,200 rpm (Shandon Cytospin, Pittsburgh, PA) onto glass slides and stained by the Diff-Quik blood stain (Baxter Scientific, Miami, Fla); the supernatant was removed and cells were analyzed for leukocyte subsets. The frequency of polymorphonuclear neutrophils, lymphocytes, and macrophages were morphologically evaluated, and the absolute cell number was calculated based on the total cell counts of BALF.

### 2.10. Fluorescein Isothiocyanate-Labeling (FITC) of* P. brasiliensis* Yeasts

*P. brasiliensis* yeasts were washed in phosphate buffered saline (PBS) and heat-killed at 60°C for 1 hr. Before labeling, the yeast suspension was sonicated using 3 cycles of 10 seconds each (21% amplitude) with Sonics (Vibra Cell VCX 750, Sonics & Materials) to eliminate aggregates. The yeasts were washed, adjusted to 1 × 10^6^ cells/mL in PBS, and then incubated with FITC (100 *μ*g/mL, Sigma) for 30 min. at 37°C. The yeast suspension was then washed tree times with PBS and stored at 4°C.

### 2.11. Phagocytic and Fungicidal Assays

Thioglycollate-induced peritoneal macrophages were isolated by adherence (2 h at 37°C in 5% CO_2_) to plastic-bottom tissue-culture plates (1 × 10^6^ cells/well in 24-well plates for fungicidal assays). Macrophages were washed to remove nonadherent cells and cultivated overnight with fresh complete medium (DMEM, Dulbecco's Modified Eagle's Medium, Sigma) containing 10% fetal calf serum, 100 U/mL penicillin, and 100 *μ*g/mL streptomycin in the presence or absence of recombinant IFN-*γ* (20 ng/mL in culture medium, BD-Pharmingen). For phagocytic assays, IFN-*γ* primed and unprimed macrophages were pretreated or not with MTL in the concentrations indicated in the legends for 60 min and were infected with* P. brasiliensis* yeasts labeled with fluorescein isothiocyanate (FITC) in a macrophage : yeast ratio of 1 : 1 [7, 40]. The cells were cultivated for 2 h at 37°C in 5% CO_2_ to allow fungi adhesion and ingestion. Supernatants were removed and cells were gently washed with PBS to remove any noningested or nonadhered yeasts. Cells were then harvested and the macrophages were then labeled with anti-CD11b APC (eBioscience) for 20 min at 4°C. Because* P. brasiliensis* yeast cells have a high variability in size and granularity (different sizes, number of buds, and number of nuclei), the granulocytes gates as defined by size (FSC) and granularity (SSC) to determine the macrophage population were not used. The total cells present in the samples were analyzed by flow cytometry (FACSCalibur, BD-Pharmingen). For the distinction between internalized and surface-bound yeasts (FITC-labeled* P. brasiliensis* particles), trypan blue (TB, 250 mg/mL, Sigma) was used for quenching the green surface-bound fluorescence on macrophages. TB quenching technique was performed as previously described [40]. Phagocytic assays were performed as described above and adherent/ingested cells measured using the FL1 and FL4 channels of a FACSCalibur cytometer. Cell suspensions were then treated in an ice bath with 0.1 mL of a TB solution prepared in 0.1 M citrate buffer, pH 4.0, lowering samples pH to nearly 4.0, thereby optimizing the TB quenching effect. After 1 min of incubation in ice bath, the samples were again analyzed. APC-labeled macrophages were gated, and FL1 and FL3 channels were used to discriminate ingested (green fluorescent, FL1) from adherent (red fluorescent, FL3) yeasts.

For fungicidal assays, IFN-*γ* primed and unprimed macrophages were pretreated with three different concentrations of MTL, lipoxin (Cayman Chemical, MI, USA) or N-t-BOC-methionyl-L-leucyl-phenylalanine (BocMLP), a known formyl peptide receptor antagonist (MP Biomedicals, France) for 60 min before infection with* P. brasiliensis* in a macrophage : yeast ratio of 50 : 1, and cocultivated for 2 h. It was previously verified that the employed concentrations of MTL had no adverse effects on macrophages or* P. brasiliensis* viability as determined by trypan blue and Janus Green B vital dye assays, respectively. The monolayers were then washed to remove nonadherent cells and incubated for an additional 48 h period. Plates were centrifuged (400 ×g, 10 min, 4°C), and supernatants were obtained and stored at −70°C and further analyzed for the presence of nitrite and cytokines. The wells were washed with distilled water to lyse macrophages, and suspensions were collected in individual tubes. One hundred *μ*L of cell homogenates was assayed for the presence of viable yeasts. All assays were done with five wells per condition in over three independent experiments.

### 2.12. Measurement of Eicosanoids

Lung homogenates from B10.A and A/J mice were collected as described above. Lipids were purified from filtered lung homogenate supernatants with Sep-Pak C18 cartridges according to the instructions of the manufacturer (Waters Corp., Milford, MA). Levels of eicosanoids were measured using commercial ELISA kits obtained from Neogen Corp (LXA4), Cayman Chemical Co. (PGE_2_, LTC_4_). The limit of assay detection for LTC_4_, PGE_2_, and LXA_4_ was 10 pg/mL.

### 2.13. Statistical Analysis

Data were analyzed by Student's *t*-test or two-way analysis of variance depending on the number of experimental groups using GraphPad Prism software (GraphPad Software). *p* values under 0.05 were considered significant.

## 3. Results

### 3.1. *P. brasiliensis* Infection Induces the Production of LTC_4_, LXA_4_, and PGE_2_ by Resistant and Susceptible Mice

**Figure 1:**
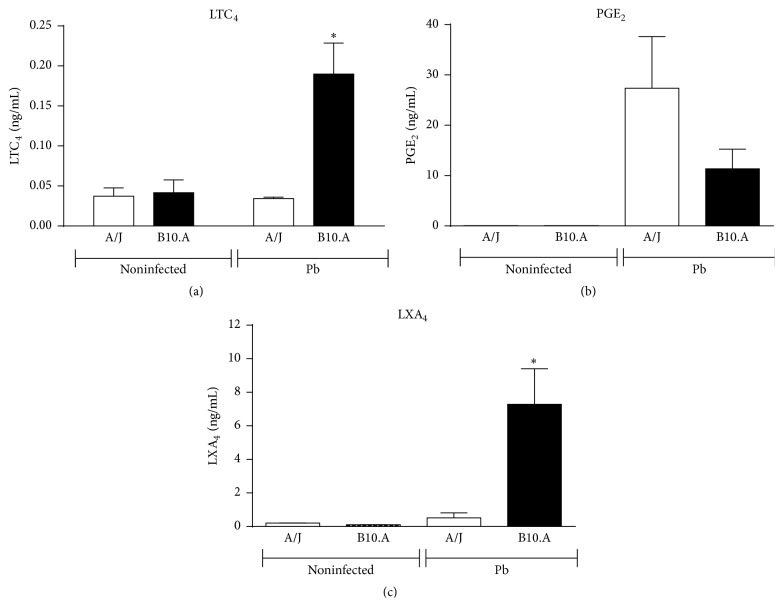
*P. brasiliensis* infection induces the production of LTC_4_, LXA_4_, and PGE_2_ by resistant and susceptible mice. Levels of lipid mediators were measured in the lung homogenates of* P. brasiliensis* (1 × 10^6^ cells) infected resistant (A/J) and susceptible mice (B10.A). The mice were sacrificed 48 h after infection and their lung homogenates assessed for the presence of LTC_4_ (a), LXA_4_ (b), and PGE_2_ (c) as detailed in* Materials and Methods*. The bars depict means ± SEM of lipid mediator levels of two experiments (5 mice per group). ^*∗*^ *p* < 0.05.

Before studying the effect of CysLT_1_ blockade in pulmonary PCM, we first asked if* P. brasiliensis* infection induces the production of lipid mediators. Thus, the levels of LTC_4_, (the major class of LTs produced in murine lungs), LXA_4_, and PGE_2_ were measured in the lung homogenates of infected resistant and susceptible mice. Susceptible (B10.A) and resistant mice (A/J) were infected with one million* P. brasiliensis* yeast cells and sacrificed 48 h later and their lung homogenates were assessed for the presence of eicosanoids.  Figure 1(a) demonstrates that* P. brasiliensis* infection induced the production of LTC_4_ by both mouse strains, but higher levels were found in the lungs of susceptible mice. LXA_4_ and PGE_2_ were also detected in 48 h lung homogenates (Figures 1(b) and 1(c)). Compared with resistant mice, increased levels of LXA_4_ (Figure 1(c)) were found in the lungs of susceptible mice, but no differences in PGE_2_ levels were detected (Figure 1(b)). Thus, 5-LO-derived products were more prominent in the lungs of susceptible mice.

### 3.2. Blockade of CysLTR_1_ Induces a Less Severe Pulmonary Infection in Susceptible, but Not in Resistant Mice

**Figure 2:**
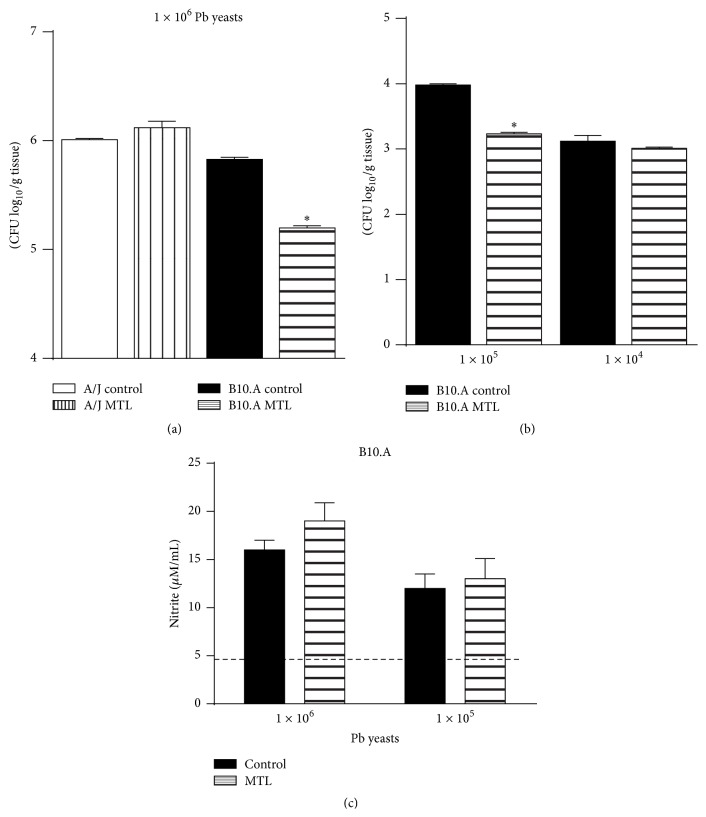
Blockade of CysLTR_1_ induces a less severe pulmonary infection in susceptible but not in resistant mice. 1 × 10^6^ *P. brasiliensis* yeasts were inoculated into A/J and B10.A mice treated or not with montelukast (MTL) during 3 days (10 mg/kg/day by the oral route) starting 24 h before infection. The severity of infection was assessed 48 h later by determining fungal loads (CFU) in the lungs (a). The severity of infection was also assessed in B10.A mice treated or not with MTL and infected with 1 × 10^5^ and 1 × 10^4^ *P. brasiliensis* yeasts (b). The levels of NO in the lung homogenates of MTL-treated and untreated B10.A mice infected with 1 × 10^6^ and 1 × 10^5^ *P. brasiliensis* yeasts were measured by Griess reagent. The dashed line indicates the levels +/− 2 SD of NO levels measured in the lungs of untreated, noninfected mice (c). Data are means ± SEM of three experiments, ^*∗*^ *p* < 0.05.

To assess the effect of CysLTR_1_ blockade in the severity of infection, an inoculum of 1 × 10^6^ *P. brasiliensis* was injected into A/J and B10.A mice treated or not with MTL during 3 days (10 mg/kg/day by the oral route) starting 24 h before infection and fungal burdens measured 48 h after infection. As can be seen in Figure 2(a), decreased numbers of viable* P. brasiliensis* yeasts were recovered from lungs of MTL-treated susceptible mice while no differences were noticed between MTL-treated and untreated resistant mice. This effect of MTL treatment was also observed with a lower infecting dose (1 × 10^5^ yeast cells) but not with 1 × 10^4^ cells (Figure 2(b)). We have further measured the levels of nitric oxide (NO) in the lung homogenates of MTL-treated and untreated B10.A mice infected with 1 × 10^6^ and 1 × 10^5^ *P. brasiliensis* yeasts. The levels of NO detected were proportional to the infecting dose employed, but no differences were noticed between MTL-treated and untreated groups (Figure 2(c)). Thus, the reduced fungal loads observed in MTL-treated mice could not be ascribed to increase NO production.

### 3.3. Montelukast Treatment Alters the Expression of ALOX5 and ALOX15 Genes

**Figure 3:**
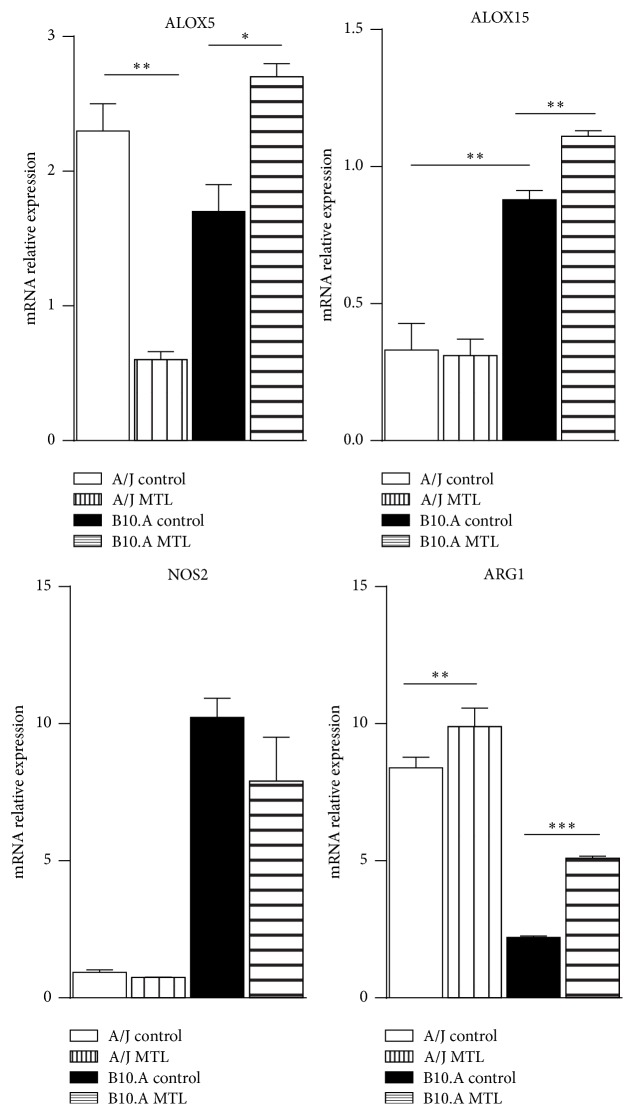
Blocking of CysLTR_1_ alters the expression of ALOX5, ALOX15, NOS2, and ARG1 mRNA. Quantitative PCR analysis of ALOX5, ALOX15, NOS2, and ARG1 genes expression. A/J and B10.A mice were untreated or treated with montelukast (MTL) during 3 days (10 mg/kg/day by the oral route) starting 24 h before infection and infected with 1 × 10^6^ *P. brasiliensis* yeasts. Total RNA from whole lungs was obtained 48 h after infection, reverse transcribed, and cDNA amplified. Real-time PCR was performed using TaqMan universal master mix. Amplified products were normalized to the amount of GAPDH products. Data represent the means ± SEM of at least 5 mice/group and were obtained in two independent experiments. (^*∗*^ *p* < 0.01 and ^*∗∗*^ *p* < 0.05).

Because 5-LO and 15-LO enzymes are involved in the synthesis of LXs, we decided to assess the influence of MTL treatment in the expression of their genes (ALOX5 and ALOX15, resp.) by* P. brasiliensis* infected mice. Therefore, MTL-treated and untreated A/J and B10.A mice were infected as previously described, and the expression of ALOX5 and ALOX15 mRNA was measured by RT-PCR. As shown in Figure 3,* P. brasiliensis* infection induced equivalent levels of ALOX5 mRNA in B10.A and A/J mice; however, MTL treatment reduced the expression of ALOX5 in the lungs of A/J mice whereas increased mRNA levels were observed in B10.A mice. Importantly, higher levels of ALOX15 mRNA were detected in the lungs of infected B10.A than A/J mice, and only in B10.A mice MTL treatment increased ALOX15 mRNA expression.

Since LXs have antiflogistic effects on inflammatory reactions, we have also measured the expression of NOS2 and ARG1 mRNA, respective gene markers of pro- and anti-inflammatory macrophages. As we have previously shown [6, 7], infected B10.A mice showed increased expression of NOS2 whereas A/J mice predominantly expressed ARG1 mRNA. MTL treatment did not significantly alter NOS2 but increased ARG1 mRNA expression in both mouse strains.

### 3.4. Blocking of CysLTR_1_ Reduces PMN Influx, Production of Proinflammatory Cytokines, and Expression of Some Pattern Recognition Receptors by Macrophages from Lungs of* P. brasiliensis* Infected Mice

**Figure 4:**
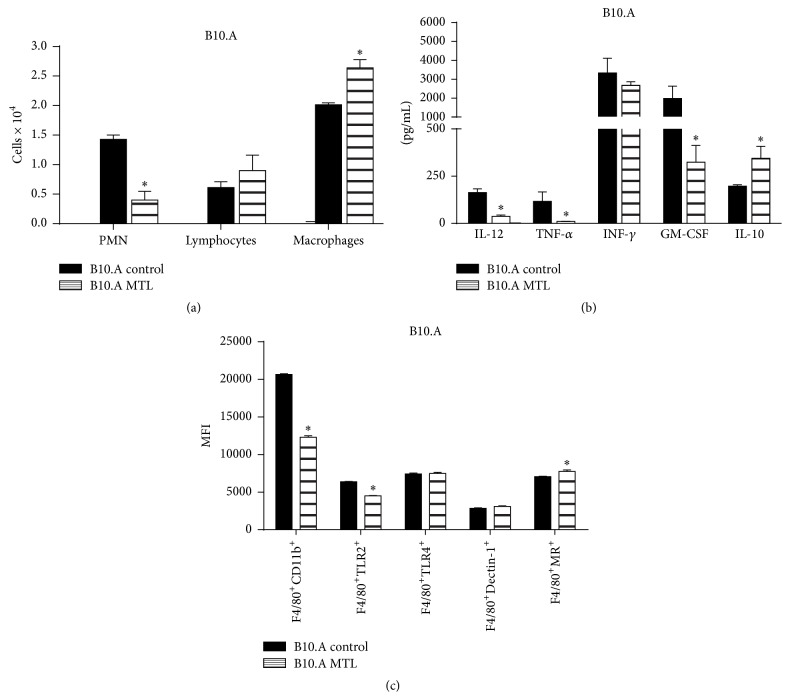
Blocking of CysLTR_1_ alters the pulmonary inflammatory exudates, the production of cytokines, and the expression of pattern recognition receptors (PRRs) by* P. brasiliensis* infected mice. MTL-treated and untreated B10.A mice were infected with 1 × 10^6^ *P. brasiliensis* yeasts and the presence of (a) inflammatory cells (macrophages, PMN neutrophils, and lymphocytes) in the bronchoalveolar lavage fluid was analyzed 48 h later (5-6 mice per group). The cell suspensions were obtained and cytospun onto glass slides. Cells were stained by the Diff-Quick blood stain. (b) The levels of pulmonary cytokines were measured in lung cell homogenates of MTL-treated and untreated B10.A mice infected with 1 × 10^6^ *P. brasiliensis* yeasts. 48 h after infection, lungs were collected, disrupted in 5.0 mL of PBS, and supernatants analyzed for cytokine content by capture ELISA. (c) Median fluorescence intensity (MFI) of PRRs (CD11b, TLR2, TLR4, Dectin-1, and MR) expressed by F4/80^+^ lung leukocytes 48 h after* P. brasiliensis* infection of B10.A mice. The lung and leukocyte suspensions were obtained and stained as described in materials and methods. Cells were analyzed by flow cytometry, and the acquisition and analysis gates were restricted to F4/80 labeled macrophages. The results are depicted as means ± SEM of two experiments (5-6 mice per group). ^*∗*^ *p* < 0.05.

Our previous studies demonstrated that, in comparison with resistant mice, susceptible mice develop a higher inflammatory cell influx to the lungs peaking at 48 h after infection and this difference was mainly due to increased migration of neutrophils to the lungs [47]. To study the effect of MTL treatment in the early inflammatory response, MTL-treated and untreated B10.A mice were infected with one million* P. brasiliensis* yeasts and the presence of inflammatory cells in the bronchoalveolar lavage fluid was analyzed 48 h later. MTL administration caused a decreased influx of PMN leukocytes to the alveolar spaces, associated with increased presence of macrophages (Figure 4(a)).

To better characterize the effect of CysLTR_1_ blockade in the inflammatory response of B10.A mice, the levels of pulmonary cytokines were measured in lung cell homogenates of MTL-treated and untreated B10.A mice (Figure 4(b)). 48 h after infection, the levels of IL-12 (p70), TNF-*α* and GM-CSF were found significantly diminished in MTL-treated mice. No differences were detected in IFN-*γ* levels, although MTL treatment resulted in increased levels of IL-10. Thus, blockade of CysLT signaling induced an enhanced anti-inflammatory response that was associated with diminished fungal loads in the lungs.

Because the anti-inflammatory activity of LXA_4_ was previously reported to reduce the expression of complement receptor 3 (CR3), the main *β*2 integrin (CD11b/CD18) expressed by leukocytes [48], we decided to evaluate the expression of CD11b and other pathogen recognition receptors [toll-like receptor 2 (TLR2), TLR4, Dectin-1, and mannose receptor (MR)] by F4/80^+^ pulmonary macrophages of MTL-treated and untreated B10.A mice (Figure 4(c)). A decreased expression of CD11b and TLR2 concomitant with increased expression of MR was observed in macrophages of MTL-treated mice, suggesting the development of a prevalent anti-inflammatory milieu.

### 3.5. Inhibition of CysLT Signaling Does Not Alter the Chronic Phase of* P. brasiliensis* Infection but Genetic Deficiency of 5-LO Results in Less Severe Disease

**Figure 5:**
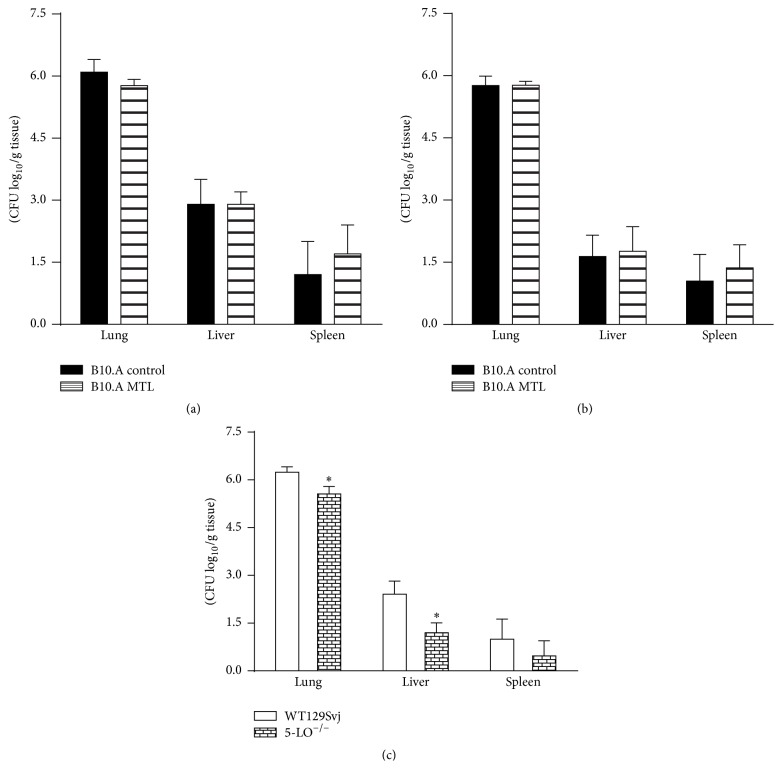
Inhibition of CysLT signaling does not alter the chronic phase of* P. brasiliensis* infection, but 5-LO deficiency results in less severe disease. MTL was administered by oral route in the dose of 10 mg/kg from day −1 until day 10 (a) or from day −1 until day 56 (b) after infection with 1 × 10^6^ yeast cells, and B10.A mice were sacrificed at week 8 after infection. The severity of infection was evaluated by determining fungal loads (CFU assays) in the lungs, livers, and spleens. The effect of LTs production was also studied using mice genetically deficient in the enzyme 5-LO. The recovery of fungal loads (CFU) from organs of 5-LO^−/−^ and control WT129Svj mice was measured 8 weeks after infection (c). The bars represent mean ± SEM log_10_ numbers of CFU obtained from groups of 5-6 mice. The results were obtained in two independent experiments. ^*∗*^ *p* < 0.05.

To determine the effect of CystLT_1_ blockade in the late infection caused by* P. brasiliensis* infection, two protocols of MTL treatment were tested. In the first, MTL was administered by oral route in the dose of 10 mg/kg from day −1 until day 10 after infection with 1 × 10^6^ yeast cells and animals were sacrificed at week 8 after infection. In the second, mice were treated by oral route with 10 mg/kg during the whole course of infection. Both protocols did not affect the fungal burdens of B10.A (Figures 5(a) and 5(b)). We have further investigated the effect of LTs production in the severity of pulmonary PCM developed by 5-LO genetically deficient mice. No differences in fungal loads were observed at week 2 after infection (5.32 ± 0.37 and 5.68 ±0 .14 log_10_ CFU in the lungs of WT and 5-LO KO mice, resp.), but at week 8 decreased CFU counts were detected in the lungs and livers of 5-LO deficient mice when compared with WT controls (Figure 5(c)).

### 3.6. Blockade of CysLTR_1_ Reduces the Fungal Loads Recovered from* P. brasiliensis* Infected Macrophages

**Figure 6:**
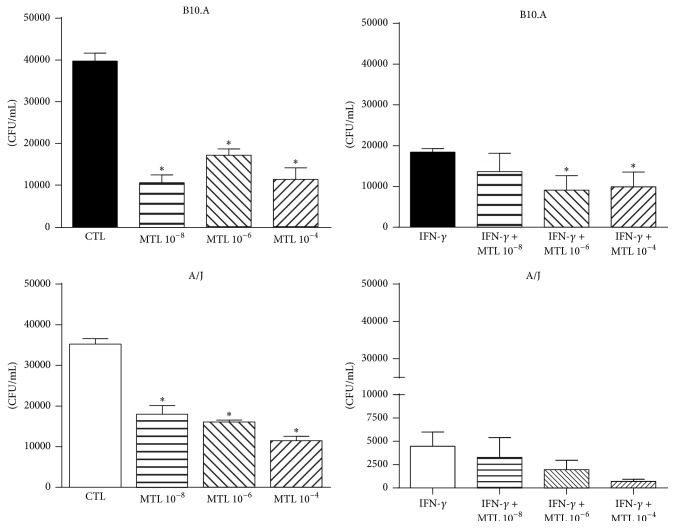
Blockade of CysLTR_1_ reduces the fungal loads recovered from* P. brasiliensis* infected macrophages. MTL (10^−8^, 10^−6^, and 10^−4 ^M) treated IFN-*γ* primed (20 ng/mL) and unprimed peritoneal macrophages from B10.A, and A/J mice were infected with* P. brasiliensis* in a macrophage-yeast cells rate of 50 : 1. After 48 h of cocultivation, the monolayers were lysed and assayed for the presence of viable yeast cells by a CFU assay. Data are means ± SEM of results from quintuplicate samples from three independent experiments. ^*∗*^ *p* < 0.05.

The unusual less severe infection associated with blockade of CysLTs signaling led us to study the effect of MTL treatment in the interaction between fungal cells and macrophages. These experiments were performed by adding different doses of MTL to peritoneal macrophages that were subsequently infected with* P. brasiliensis* in a macrophage-yeast cells rate of 50 : 1 [6]. The diverse doses of MTL employed were able to reduce the fungal loads of IFN-*γ* primed and unprimed B10.A macrophages. However, in A/J cells, a significant reduction in CFU counts was only observed in unprimed cells (Figure 6). This result reproduces our previous in vivo findings with B10.A mice and demonstrates that the in vitro killing ability of A/J phagocytes was also affected by MTL treatment.

### 3.7. MTL Treatment Reduces the Phagocytic Ability of Macrophages

**Figure 7:**
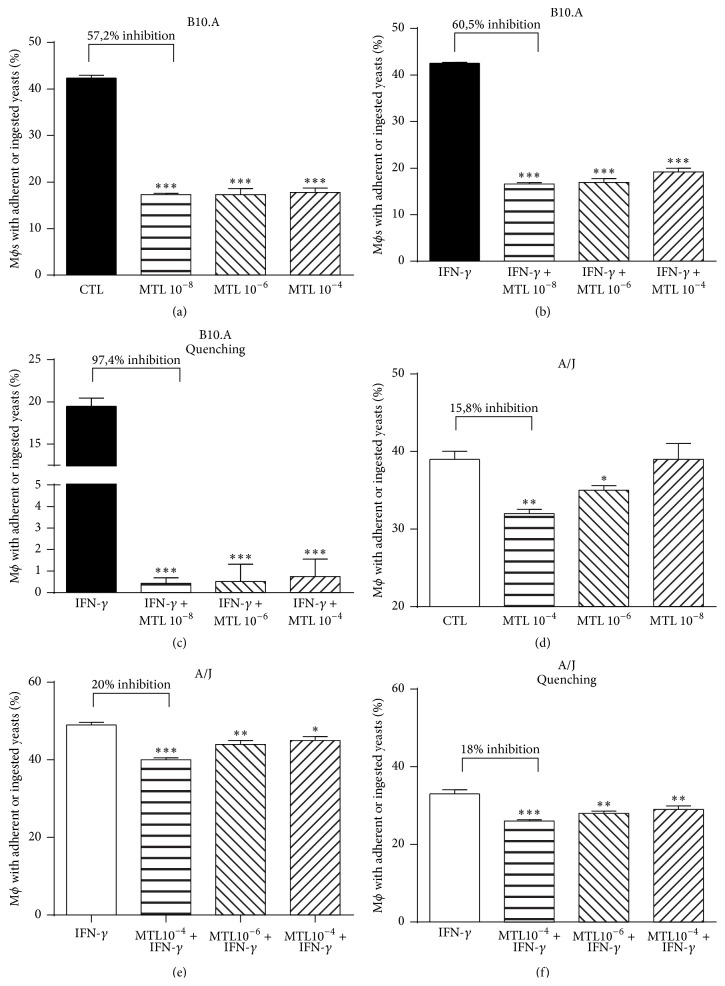
MTL treatment reduces the phagocytic ability of macrophages. For phagocytic assays, IFN-*γ* (20 ng/mL) unprimed (a, d) and primed (b, c, d, and f) macrophages from B10.A and A/J mice were pretreated or not with three concentrations (10^−8^, 10^−6^, and 10^−4 ^M) of MTL. The macrophages were then infected FITC-labeled* P. brasiliensis* at macrophage-yeast ratio of 1 : 1 for 2 h at 37°C to allow fungi adhesion and ingestion. Macrophages were washed; cells detached from plastic and were labeled with anti-CD11b APC antibodies. The cell suspensions were immediately read on a FACSCalibur cytometer (a, b, d, and e). A quenching assay employing a trypan blue solution (TB: 250 *μ*g/mL) was used to distinguish internalized from surface-bound yeast (c, f). Data are means ± SEM of results from quintuplicate samples from three independent experiments. ^*∗∗∗*^ *p* < 0.001.

The unexpected reduced fungal loads induced by CysTL_1_ blockade led us to ask whether this difference was due to decreased fungal uptake or increased killing ability of macrophages. We employed a phagocytic assay that allowed us to distinguish adherent/ingested from ingested yeast cells. To this end, the interaction of PI-labeled* P. brasiliensis* with B10.A and A/J macrophages was measured before and after fluorescence quenching by trypan blue, using a cytometric assay that we have previously described [7, 40]. Macrophage cultures (1 × 10^6^/well) performed in 24-well plates were pretreated or not with three different concentrations of MTL and infected with heat-killed PI-labeled yeasts (1 : 1 fungus : macrophage ratio). After 2 h of incubation, supernatants were aspirated, the monolayer was gently washed with PBS, and the cells were harvested. To determine the number of ingested/adhered* P. brasiliensis* yeasts, macrophages were incubated with FITC-labeled anti-CD11b and analyzed by flow cytometry. As shown in Figures 7(a) and 7(b), MTL treatment of IFN-*γ* primed and unprimed B10.A macrophages reduced by 57–60% the frequency of ingested/adhered yeasts. The quenching of extracellular fluorescence by trypan blue showed that MTL reduced by 97% the number of ingested yeasts by B10.A macrophages (Figure 7(c)). MTL-treated A/J macrophages also showed a reduced adherence (15–20%) and ingestion (18%) of* P. brasiliensis* yeasts, but this effect was much less intense than that observed in B10.A cells (Figures 7(d)–7(f)). This result suggested that the decreased fungal loads induced by CysLT_1_ blockade could be ascribed to the preferential production or signaling of inhibitory mediators of macrophage functions.

### 3.8. Exogenous Lipoxin Reduces and Blockade of Lipoxin Receptor Increases the Fungal Loads of Macrophages

**Figure 8:**
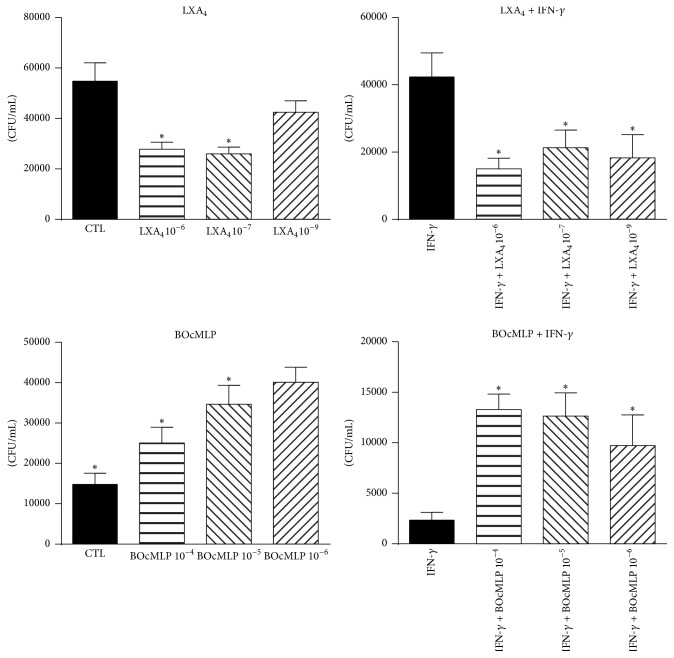
Exogenous lipoxin reduces, and blockade of lipoxin receptor increases the fungal loads of macrophages. IFN-*γ* primed (20 ng/mL) and unprimed macrophages from B10.A mice were treated with several doses of synthetic lipoxin (10^−9^, 10^−7^, and 10^−6^ M) or a specific antagonist of lipoxin receptor (BOcMLP) (10^−5^, 10^−6^, and 10^−4^ M) and infected with* P. brasiliensis* in a macrophage-yeast cells rate of 50 : 1. After 48 h of cocultivation, the monolayers were lysed and assayed for the presence of viable yeasts by a CFU assay. Data are means ± SEM of results from quintuplicate samples from three independent experiments. ^*∗*^ *p* < 0.05.

To further characterize the effect of lipoxin and its receptor in the interaction between macrophages and* P. brasiliensis* yeasts, IFN-*γ* primed and unprimed macrophage cultures were treated with several doses of synthetic lipoxin or a specific antagonist of lipoxin receptor (BOcMLP). As depicted in Figure 8, the addition of exogenous lipoxin significantly reduced the fungal burden of IFN-*γ* primed and unprimed B10.A macrophages. Furthermore, the blockade of lipoxin receptor increased the fungal loads of macrophages (Figure 8). These results led us to infer that lipoxin production and signaling were able to reduce the severity of infection caused by* P. brasiliensis* yeasts.

## 4. Discussion 

The role played by LTs in murine paracoccidioidomycosis is still controversial. Pharmacological inhibition of LT synthesis employing the FLAP inhibitor MK 886 [35] showed a protective effect of LTB_4_ in the early pulmonary infection developed by two mouse strains selected for high and low inflammatory responses [35]. However, two recent reports using 5-LO deficient C57BL/6 mice reported opposite results. In intravenously infected mice, absence of 5-LO activity was shown to be protective [34], whereas in intratracheally infected mice a deleterious effect was observed [36]. According to the former report [34], 5-LO sufficient mice secrete elevated and sustained levels of LX not observed in 5-LO^−/−^ mice that develop a less severe disease and enhanced Th1 immunity. In contrast, Santos et al. [36] showed an early mortality of 5-LO^−/−^ mice accompanying an early deficiency of LTB4 production. These discrepant results led us to further investigate the role of LTs in i.t. infected mice, a route that better mimics the human disease. Here, we decided to investigate the role of CysLTs since (1) they are less studied than LTB_4_ during host defense and (2) murine lung cells produced ~10 times more CysLTs than LTB_4_. Using resistant and susceptible mice to* P. brasiliensis* infection, we first verified that the genetic pattern of hosts influence the synthesis of PGE_2_, LTC_4_, and LXA_4_. Compared with resistant mice, an increased production of LTC_4_ and LXA_4_ by susceptible B10.A mice was observed 48 h after i.t. infection. Consistent with these data, susceptible mice expressed higher levels of 15-LO mRNA than A/J mice possibly explaining their elevated production of LXA_4._ Furthermore, MTL treated B10.A mice increased the mRNA expression of 5-LO and 15-LO mRNA, conceivably increasing the synthesis of LXs and their inhibitory activities on inflammatory reactions.

Using in vitro and in vivo models of infection, we could demonstrate for the first time the important role of CysLTs signaling in pulmonary paracoccidioidomycosis. Interestingly, and consistent with the levels of eicosanoids produced, inhibition of CysLT_1_ receptor by MTL treatment modulated the early in vivo infection of susceptible but not resistant mice. A low number of viable yeasts were recovered from lungs of B10.A mice 48 h after infection, and this result was not associated with the increased NO levels, the most important fungicidal molecule of macrophages [40, 49, 50], indicating that NO-independent mechanisms were responsible by the less severe* P. brasiliensis* infection. Our results have also suggested that the LXA4/LTC4 ratio has an important influence in the fate of infection. Thus, 48 h after* P. brasiliensis* infection of A/J mice, the LXA4/LTC4 ratio was 1.0, whereas in B10.A mice it was 37.5, possibly explaining the LXA4-governed phenotypes observed only in the susceptible mouse strain.

At the chronic phase of infection, MTL treatment did not influence the severity of the disease indicating a prevalent action of CysLTs at the innate phase of immunity. In contrast, decreased fungal loads were observed in 5-LO^−/−^ mice only at week 8 of infection, revealing that the concomitant deficiency of LT and lipoxin production affects the fungal burdens controlled by the adaptive phase of immunity. These findings support those reported by Tristão et al. [34], but they are in contrast with those of Santos et al. [36] that postulated a prevalent role of 5-LO activity and LTs synthesis at the innate phase of immunity [34, 36].

LTs are involved in the recruitment and activation of inflammatory cells and play a relevant role in the control of several inflammatory, infectious, and neoplastic diseases [14, 51–53]. In contrast, LXA_4_ and ATL (aspirin-triggered lipoxin) behave as potent negative regulators of inflammation due their ability to inhibit PMN neutrophil and eosinophil traffic. These effects are achieved by enhancing PMN clearance from inflammatory focus without excessive macrophage activation, modulating cytokine-stimulated metalloproteinase activity and inhibiting cell proliferation [16]. LXA_4_ production was also involved in the prevalent synthesis of anti-inflammatory cytokines such as IL-10 that play a fundamental role in the resolution of inflammation [54, 55]. Here, we have verified that MTL-treated B10.A mice displayed several features of prevalent LX signaling. Indeed, the low influx of PMN cells to the site of infection and the decreased production of TNF-*α* and IL-12 associated with enhanced production of IL-10 detected 48 h after infection of MLT-treated B10.A mice indicated a prevalent LX signaling when CysLTR_1_ was blocked. The low secretion of proinflammatory cytokines associated with the increased production of IL-10 demonstrated a shift of the initial host response toward an anti-inflammatory milieu. This anti-inflammatory response concomitant with diminished fungal loads is an unusual finding once the efficient control of* P. brasiliensis* growth is mediated by proinflammatory cytokines and activated phagocytes [5, 6, 40, 47, 56]. Furthermore, the mild or regressive forms of paracoccidioidomycosis have been associated with decreased or absence of IL-10 production, respectively [57, 58]. Despite these observations, we believe that in our in vivo studies the less severe pulmonary infection of MTL-treated B10.A mice could be ascribed to the improved mechanisms of innate immunity induced by the increased levels of lipoxin synthesized by this mouse strain at the acute phase of infection. Besides its potent anti-inflammatory activity, LXA4 can also exert protective effects in lung infections by increasing pathogen clearance mediated by mucociliary activity of airways. Indeed, in cystic fibrosis, a pathology associated with decreased LXA_4_ synthesis, administration of a stable analog of LX leads to regulated ion transport and restored airway surface liquid height via the ALX/FPR2 receptor, which is expressed by epithelial cells [59, 60]. The restored airway hydration increases the mucociliary clearance of pathogen contributing to the improved resolution of inflammation mediated by lipoxin. Our model is tempting to suppose that the decreased phagocytic ability of macrophages resulted in increased numbers of extracellular fungal cells that were better eliminated by an improved mucociliary activity. In addition to their anti-inflammatory activity, LXs are also host protective by improving mucosal bacterial killing via expression of bacterial/permeability inducing protein (BPI) in epithelial cells [61].

Our additional studies with in vitro infected macrophages recapitulated our in vivo findings and demonstrated that the decreased ingestion of* P. brasiliensis* yeast by inflammatory phagocytes could account, at least partially, for the reduced fungal loads. Importantly, the phagocytic assays using PI-labeled* P. brasiliensis* yeasts explained the low CFU counts observed in the fungicidal analyses and suggested that the CysLTR_1_ blockade resulted in impaired interaction between macrophages and fungal cells. Moreover, the in vitro treatments of macrophages with exogenous lipoxin and an antagonist of lipoxin receptor demonstrated that CysLTs exert an activating effect, whereas lipoxin inhibits the interaction between macrophages and* P. brasiliensis* yeasts. This impaired interaction could be explained by the decreased expression of pathogen recognition receptors (PRRs) by lipoxin-stimulated macrophages [48, 62, 63]. Our previous studies have demonstrated that the absence of TLRs expression or MyD88 signaling results in decreased interaction of macrophages with* P. brasiliensis* cells [9, 37, 38, 56]. We have also reported that CR3 (CD11b/CD18) plays a fundamental role in the interaction of B10.A macrophages with* P. brasiliensis* yeasts, in contrast with A/J macrophages that preferentially use Dectin-1 and MR to sense* P. brasiliensis* infection [7]. Here, a decreased expression of CD11b and TLR2 was observed following MTL treatment possibly contributing to the inhibited recognition of* P. brasiliensis* by B10.A macrophages. Interestingly, lipoxin was also shown to exert an anti-inflammatory effect in periodontal disease caused by* Porphyromonas gingivalis* by impairing the expression of CR3 [48].

Previous studies with resistant and susceptible mice to* P. brasiliensis* infection have demonstrated that an elevated activation of innate immunity does not necessarily lead to less severe infections or better disease outcomes [6–9, 37, 38]. Early after* P. brasiliensis* infection, susceptible B10.A mice mount a more vigorous, PMN-rich, proinflammatory reaction than resistant A/J mice [47]. An increased killing ability associated with elevated IL-12 and NO secretion is a feature of alveolar macrophages and DCs from susceptible mice, whereas a preferential secretion of TGF-*β* impaired inflammatory and fungicidal activities, the behavior of A/J cells. Thus, the production of proinflammatory mediators such as IL-12, TNF-*α*, IFN-*γ*, and LTs is essential to control microbial growth during infection, but their effects have to be regulated by anti-inflammatory mechanisms and mediators to avoid excessive tissue destruction and suppression of adaptive immunity as what occurs with susceptible mice [11, 58]. Murine cerebral malaria, toxoplasmosis, and tuberculosis are other examples of infections where excessive proinflammatory response results in severe tissue pathology that, however, is counter-regulated by lipoxin production [24, 25, 64]. In sum, a balanced and efficient immunity to a microbial infection must express a proinflammatory activity to control pathogen growth, but this activity must be tightly regulated by anti-inflammatory mechanisms to guarantee the integrity of host tissues.

In pulmonary paracoccidioidomycosis, the protective effect of CysLTR_1_ blockade that results in less severe infection was not previously described. Furthermore, prevalent lipoxin secretion and signaling that have been previously described as an escape mechanism of pathogens [24] was here associated with host immunoprotection. In pulmonary paracoccidioidomycosis of susceptible B10.A mice, whose susceptibility is based in the early excessive proinflammatory responses, the inhibitory effect of lipoxin signaling could exert a beneficial effect. Importantly, even in human paracoccidioidomycosis the excessive activation of the immune system can be deleterious, and in some cases an effective therapy is only achieved if the antifungal treatment is associated with anti-inflammatory drugs [65, 66].
